# An overview of Indian research in personality disorders

**DOI:** 10.4103/0019-5545.69241

**Published:** 2010-01

**Authors:** Pratap Sharan

**Affiliations:** Department of Psychiatry, All India Institute of Medical Sciences, New Delhi, India

**Keywords:** Personality disorders, research, epidemiology

## Abstract

Personality disorders have significant, but often unrealized, public health importance. The present review summarizes the published work on personality disorders in the Indian population or by Indian researchers residing in the country. Researchers who have worked on assessment methodology in India have demonstrated that clinical diagnosis has a low reliability when compared with semi-structured interviews; and have attempted to increase the feasibility of the standardized use of International Personality Disorder Examination, a semi-structured interview developed by the World Health Organization (WHO). Studies on epidemiology demonstrate that none of the general population studies have employed standardized interviews, and hence, they grossly underestimate the prevalence of personality disorders in the community. The clinical epidemiology studies have employed questionnaires and interviews developed in the West, mostly without local adaptations, with discrepant results. However, these studies show that personality disorders are common in the clinical population and that rates vary across sub populations. While, there are a few reports attesting the theoretical importance of the role of culture in the formation and expression of personality disorders, empirical literature from India in this area is scanty. Similarly, there are few reports on the treatment of personality disorders, while, important areas such as service delivery, etiology, and validity of personality disorders, are unaddressed. The study of personality disorder in India is maturing, with researchers showing increased familiarity with the methodological nuances of this complex area of research.

## INTRODUCTION

The definition of personality disorders given by the International classification of diseases (ICD-10) states that ‘personality disorders’ comprise of deeply ingrained and enduring behavioral patterns, manifesting themselves as inflexible responses to a broad range of personal and social situations. They represent extreme or significant deviation from the manner in which an average individual in the given culture perceives, thinks, feels, and particularly relates to others. They are frequently, though not always, associated with varying degrees of subjective distress and problems in social functioning and performance. These patterns are usually evident during late childhood or adolescence, but the requirement to establish their stability and persistence usually (but not necessarily) restricts the use of the term ‘disorder’ for adults.[1] The Diagnostic and Statistical Manual of Mental Disorders IV (DSM-IV) definition is similar, although it is more explicit, and emphasizes the impulse control problems that many patients with personality disorders would have.[2]

Personality disorders lead to a disturbance in functioning as great as that in most major mental disorders.[3] They are associated with high rates of separation and divorce; unemployment and inefficiency; and poor quality of life for the individual and his/her family. Patients with personality disorders have an increased risk of mortality through suicide, homicide, and accidents. Moreover, when a personality disorder is present, the treatment of other coexisting psychiatric or medical conditions is frequently more complicated, lengthier, or less successful; a pattern that may at times be due to lack of recognition of the personality disorder.[4–6]

Since the publication of the Diagnostic and Statistical Manual (DSM-III) in 1980, and its creation of a separate diagnostic axis (i.e., Axis II) for personality disorders, interest in the description and classification of personality disorders has expanded dramatically in the West. The present review summarizes the published work on personality disorders in the Indian population or by Indian researchers residing in the country. It excludes studies on normal personality variants and studies that use personality measures in relation to other foci of interest.

## STUDIES ON HISTORY

Neki states that the proposition that human beings are different in their personality style evolved from antiquity.[7] Charaka applied the Tri-guna theory to the clinical situation and postulated that man’s prakriti (nature) was defined by the relative accentuation of any of the three gunas: Satvik (consciousness), rajsik (energy) or tamsik (inertia).

## STUDIES ON ASSESSMENT METHODOLOGY

The field of personality disorders is beset with problems of reliability and validity. The reliability of the personality disorder diagnoses, when assessed without diagnostic instruments is lower than state disorder (Axis I) diagnoses. Furthermore, the reliability across personality disorder instruments, particularly between self-report instruments and semi-structured interviews; is low, such that self-report instruments are considered good only for screening personality disorders.[8] Naidu and Issac found that only 36.3% of the psychiatric inpatients, diagnosed to have a personality disorder clinically, also had a personality disorder diagnosis according to a semi-structured interview schedule (SCID II), attesting to the low reliability of clinical diagnosis.[9] However, the authors did not specify whether SCID II was standardized for use in India. This is relevant because some unreliability could have been introduced by difficulties in cross-cultural application of a Western instrument.

A number of assessment instruments for the diagnosis and measurement of personality disorders are now available for clinical and research use in the West. Some of these focus on individual personality disorders such as schizotypal, borderline or depressive, while others are comprehensive in their coverage. Among the latter, both self-rating instruments useful for screening purposes, such as, Personality Disorder Questionnaire (PDQ)[10] and Millon Clinical Multiaxial Inventory (MCMI)-III;[11] and semi-structured interview-based instruments, such as, Structured Clinical Interview for DSM-III R Axis II (SCID-II)[12] and International Personality Disorder Examination (IPDE) are available.[13] The latter provides ICD-10 diagnoses. Although it has good psychometric properties, its length makes it difficult to use in the community, in population research, particularly outside the psychiatric settings. Mann *et al*., evaluated the utility of the informant-based Standard Assessment of Personality (SAP) as a screen for the International Personality Disorder Examination (IPDE), in out-patients in Bangalore.[14] The overall agreement between the two instruments in the detection of ICD-10 personality disorder was modest (kappa = 0.4). The level of agreement varied according to the personality disorder category, ranging from kappa 0.66 (dependent) to kappa 0.09 (dyssocial). The SAP proved to have a high negative predictive value (97%) for IPDE as the gold standard, suggesting its potential as a screen in samples where the expected prevalence of personality disorder was low. However, Loranger *et al*. have developed a screening questionnaire for the IPDE, therefore, the relative advantage of the SAP as a screen for IPDE needs to be re-established.[13]

Although SAP and IPDE screening questionnaires address the issue of brevity, they are not standardized for use in the local languages of India. Sharan *et al*., translated the ICD-10 IPDE into Hindi following a standard translation protocol, and established the joint rater reliability and applicability of the Hindi version in non-psychotic adult outpatients.[15] The average intraclass correlation of each item (0.89), the number of criteria met per disorder (0.92), and the dimensional scores (0.98) were high. Kappa for definite (0.65 to 0.78) and probable (0.78 to 1.00) personality disorder and for the presence/absence of any personality disorder (0.78) was acceptable. The overall weighted kappa was 0.81 for definite and 0.91 for probable personality disorder. The patients experienced difficulty in understanding questions related to self-image, internal preferences, emptiness, and emotional shallowness. Further, the interviewers faced difficulty in scoring certain items because of cultural variation in what can be considered a norm, for example, the avoidance of occupational behaviors that involve interpersonal contact (difficult to label abnormal in housewives), encouraging/allowing others to make important decisions (difficult to label abnormal in many women/young adults), unwillingness to become involved with persons unless they were certain of being liked (difficult to label abnormal in persons who miss out on relationships with members of the opposite sex), hitting family members (difficult to label abnormal as norm varies); lying (difficult to label abnormal as no norm is specified), and speeding/reckless driving (difficult to label abnormal in those who do not own a vehicle). However, overall, the results suggested that ICD-10 IPDE (Hindi Version) has acceptable joint rate reliability and applicability in the North Indian Hindi speaking population.

## STUDIES ON EPIDEMIOLOGY

### Community studies

Four general population studies done in the late 1980s and early 1990s that used assessment instruments specific to personality disorders established the high (and consistent) prevalence of these disorders (10.3 to 13.5%) in developed countries.[16] More recent studies have upheld these results. The sex ratio was different for specific types of personality disorders, although the overall rate of prevalence was roughly equal for the two sexes.[17] Reddy and Chandrashekar conducted a meta-analysis of 13 epidemiological studies from different parts of India.[18] The prevalence of personality disorders was assessed in seven studies and the rate varied from 0 to 2.8%, with the weighted prevalence rate being 0.6%. Personality disorder diagnosis was significantly associated with the male gender. Prevalence rates of personality disorders may be lower in developing countries, but the methodological shortcomings of surveys preclude direct comparisons with the western data. Most of the general population epidemiological studies conducted in India have neglected co-morbidity and dual diagnosis, and have used screening instruments with low sensitivity and single informants; hence, they systematically underreport the prevalence.[19]

### Clinical studies

Early studies (that did not employ diagnostic instruments or operationalized criteria) on clinical samples from India reported prevalence rates of 0.3-1.6%.[20–23] However, the rates were higher in special populations such as university students (19.1%);[22] criminals (7.3-33.3%);[24–26] patients with substance use disorders (20-55%);[27–29] and patients who attempted suicide (47.8-62.2%).[30,31] Studies employing comprehensive protocols for assessment (which were, however, not standardized for use in the Indian population) have yielded high rates of personality pathology in patients with anxiety disorders, such as, social phobia;[32] drug dependence (25.6%);[33] and mood disorders (37.5% in patients with bipolar disorder and 40.8% in those with major depressive disorder).[34] The study on mood disorders used a self-report format for assessing personality disorders, which is known to overestimate the prevalence of these disorders.[34]

In the International Pilot Study of Personality Disorders (IPSPD), the following personality disorders were frequently diagnosed in the clinical sample at Bangalore: Schizotypal (19.1%) and borderline (14.7%) according to the DSM-III-R system; and emotionally unstable (8.6%) according to the ICD-I0 system.[13] Banerjee and Mitra compared 50 teenage girl outpatients with academic difficulties with normal controls.[35] About 30% of the index group had emotionally unstable personality disorder (impulsive type), 6% had dependent personality disorder, and 6% other personality disorders, according to the ICD-10. The authors did not report whether they used the IPDE screen or the interview for diagnosing personality disorders.

### Studies in relation to self-harm

The rate of personality disorders in subjects who have demonstrated acts of self-harm have varied from 7%[36] to 64%.[37] Methodological issues probably play a major role in the discrepancy of prevalence rates between studies. Nath *et al*., used the International Personality Disorder Examination (IPDE) to assess outpatients and inpatients, who presented with a history of self-harm at any point in their life, in two age groups (15-24 years and 45-74 years).[37] Sixty-four percent of the older group and 58.5% of the young subjects were reported to have a personality disorder. In the young group the most common personality disorder was the emotionally unstable personality disorder (28.6%) and anankastic personality disorder (11.7%); while in the older group, the anankastic personality disorder (34.5%) and emotionally unstable personality disorder (13.8%) were the most common personality disorders. The fact that all patients could be interviewed despite reluctance on the part of some (due to medico-legal concerns), and because the interview took approximately one hour (which is shorter than usual for those with a positive diagnosis), suggests that the subjects may have responded with a affirmative bias toward the questions. The fact that a local language version of the IPDE was not yet available, may also have led to some randomness in the responses. That only 5% of the young and none of the older patients had more than one personality disorder diagnosis, was surprising, in light of the high prevalence of personality disorders.

Chandrasekaran *et al*., assessed 341 survivors (93% of all survivors, over a one-year period) after their first suicide attempt from a general hospital.[36] Only 7% received a personality disorder diagnosis according to ICD 10 IPDE. The inclusion of the first attempt cases may have led to a low rate of diagnosis of emotionally unstable personality disorder (and consequently of any personality disorder). Other systematic biases could have been introduced by use of two interviewers and consensus diagnosis for all cases (the diagnostic process may have become too stringent). The article does not explicitly state it, but it is probable that the authors used the IPDE screening questionnaire for selecting subjects for the full interview; the sensitivity of the screen should have been assessed/mentioned to help in the interpretation of the findings of the study. The authors have quoted a study by Latha *et al*., which yielded a (similar) prevalence rate of 12% for personality disorders in those attempting self-harm, but the latter study reached a diagnosis without using a standardized instrument and hence the two studies are not strictly comparable.[38] A study that only assessed the presence of borderline personality disorder with a semi-structured interview, in patients who had made a suicide attempt, yielded a much higher rate of 18.3% for this single diagnosis.[39]

An interesting observation in clinical epidemiology of personality disorders in India is the relatively narrow gap in prevalence between the genders, with respect to emotionally unstable personality disorders. This could be due to the inclusion of emotionally unstable personality disorder — impulsive type in ICD 10, patterns of treatment seeking or the impact of cultural factors on the formation and expression of these disorders. An examination of the gender differential in community studies would be needed to confirm/disconfirm this interesting finding obtained in the clinical studies.

### Classification system preferred in Indian research

The ICD-10 and DSM-IV are different, but overlapping classification systems. Both have adopted a polythetic approach as against a monothetic approach, in which none of the listed criteria are essential to make a diagnosis, any combination of a required number of criteria would lead to the diagnosis. There are some differences in the nomenclatures, for example, anankastic personality disorder in ICD-10 is obsessive-compulsive personality disorder in DSM-IV. In ICD-10, schizotypal disorder is considered to be an attenuated manifestation of schizophrenia and is categorized with psychotic disorders, while, narcissistic, depressive, and passive-aggressive personality disorders do not find a mention. There are also several marked differences in the criteria of the two systems and some minor variations in the wordings. Finally, the two schemes differ, in that, DSM separates state- and trait-based disorders on two axis and provides for clusters of personality disorders; while the ICD-10 diagnostic guidelines do not place the personality disorders and state disorders on separate axis or subdivide personality disorders into clusters. It is obvious from the above-mentioned studies that ICD 10 has found greater favor with the Indian researchers, probably because of its easier application in clinical practice (retrospective studies) and greater accessibility of IPDE, as it was developed by the World Health Organization.

### Studies on cultural issues

Western authors such as Oldham state that there is little dispute about the existence of personality pathology.[4] The International Pilot Study on Personality Disorders demonstrated that disorders, as presently defined, could be identified at all sites (multinational, multilingual, multicultural).[40] However, these studies do not confirm the cross-cultural validity or usefulness of western diagnostic categories or personality dimensions, as these utilize western concepts in a non-western setting. Thus, they may have identified ethnic artifacts rather than culturally meaningful configurations.[41] Personality disorders typically carry a strong connotation of immutability that may be directly at odds with the core belief of some of the major non-western cultural traditions, which lay emphasis on the perfectability of human nature.[7] At an even more fundamental level, cross-cultural analysis has challenged concepts such as ‘person’ and ‘selfhood,’ implicity or explicitly used by theories of personality. Shweder and Bourne have described the sociocentric, holistic conception of individual society relations among Oriyas, where person units are believed to be altered by the relations into which they enter, rather than being seen as a synthesis of abstract traits.[42] Similar relational concepts of the person are reported widely in India.[43]

It has been hypothesized that culture can influence: (i) The genetic selection of specific temperamental characteristics in highly inbred groups, (ii) learnings inside and outside the family, (iii) the threshold when personality vulnerability cannot be compensated by the person (trait accentuation), and (iv) the social threshold when such decompensations are labeled pathological.[44,45] Ethnocentric work is clearly needed before the universality of personality disorders is assumed. In an article of this kind, Chowdhury and Brahma presented a case of an 18-year old man with dhat syndrome with repeated self-harm attempts initially in response to guilt caused by masturbation and voyeurism and then due to the occurrence of somatic symptoms that he associated with semen loss.[46] He was diagnosed to have borderline personality disorder according to the Self-Harm Inventory (SHI) and Personality Disorder Questionnaire-R (PDQ-R). However, more transcultural work from India is clearly required.

### Studies on management issues

Management options will depend on a large number of factors, such as, the availability of healthcare resources, the therapist’s own skill and stance, and aspects of the patient’s personality and present situation. These include his/her social support, associated psychiatric or physical illness, psychological mind-set, past relationship patterns, and areas of resourcefulness. Pradhan *et al*., presented a one-year follow up of six male borderline patients treated with pharmacotherapy and psychotherapy, who improved substantially.[47] More literature on individual management and service provision for personality disorders is urgently needed to counter the prevalent nihilism with regard to treatment outcome. Western studies suggest that many patients maintain sustained improvement with treatment.

## CONCLUSIONS

The field of personality disorders is at a nascent stage of development in India. From a situation of almost no articles specifically focused on personality pathology till the 1980s, there is now a trickle. However, to date, the focus is understandably but entirely on clinical epidemiology. Although there are very few methodologically robust studies, the increasing familiarity with the field and its methodological nuances augers well for the future. There is obviously a need for better and more studies in relation to personality disorders on methodology and epidemiology (particularly community studies), and also on cultural and classificatory issues. There is also a need for studies to populate the vast open swathes in terms of etiology, clinical features, assessment, management, course and outcome, and on the various debates that mark the personality disorder field, for example, whether personality disorders, as conceptualized today, are valid entities; the boundary issues between personality disorders and normal personality traits on the one hand and mental state disorders on the other; and the organization of personality disorder in dimensional or categorical terms.
